# An Efficient Large-Scale Retroviral Transduction Method Involving Preloading the Vector into a RetroNectin-Coated Bag with Low-Temperature Shaking

**DOI:** 10.1371/journal.pone.0086275

**Published:** 2014-01-15

**Authors:** Katsuyuki Dodo, Hideto Chono, Naoki Saito, Yoshinori Tanaka, Kenichi Tahara, Ikuei Nukaya, Junichi Mineno

**Affiliations:** Center for Cell and Gene Therapy, Takara Bio Inc. Seta, Otsu, Shiga, Japan; Emory University School of Medicine, United States of America

## Abstract

In retroviral vector-mediated gene transfer, transduction efficiency can be hampered by inhibitory molecules derived from the culture fluid of virus producer cell lines. To remove these inhibitory molecules to enable better gene transduction, we had previously developed a transduction method using a fibronectin fragment-coated vessel (i.e., the RetroNectin-bound virus transduction method). In the present study, we developed a method that combined RetroNectin-bound virus transduction with low-temperature shaking and applied this method in manufacturing autologous retroviral-engineered T cells for adoptive transfer gene therapy in a large-scale closed system. Retroviral vector was preloaded into a RetroNectin-coated bag and incubated at 4°C for 16 h on a reciprocating shaker at 50 rounds per minute. After the supernatant was removed, activated T cells were added to the bag. The bag transduction method has the advantage of increasing transduction efficiency, as simply flipping over the bag during gene transduction facilitates more efficient utilization of the retroviral vector adsorbed on the top and bottom surfaces of the bag. Finally, we performed validation runs of endoribonuclease MazF-modified CD4^+^ T cell manufacturing for HIV-1 gene therapy and T cell receptor-modified T cell manufacturing for MAGE-A4 antigen-expressing cancer gene therapy and achieved over 200-fold (≥10^10^) and 100-fold (≥5×10^9^) expansion, respectively. In conclusion, we demonstrated that the large-scale closed transduction system is highly efficient for retroviral vector-based T cell manufacturing for adoptive transfer gene therapy, and this technology is expected to be amenable to automation and improve current clinical gene therapy protocols.

## Introduction

Fibronectin (FN), one of the major extracellular matrix proteins, is a disulfide-linked dimeric glycoprotein that has several functional domains including cell binding properties [1]–[3]. FN is a glycoprotein that binds to membrane-spanning receptor proteins called integrins. In addition to integrins, FN also binds to extracellular matrix components such as collagen, fibrin, and heparan sulfate proteoglycans. A recombinant FN fragment named CH-296 [4] (RetroNectin®; RN, Takara Bio, Shiga, Japan) was found to be most effective for retrovirus-mediated gene transduction [5]–[9]. Retroviral vectors are currently one of the most widely used systems for gene transduction, both in experimental studies and in clinical trials. In particular, murine leukemia virus (MLV) has traditionally been used as the vector of choice for clinical gene therapy protocols, and a variety of packaging systems [10], [11] and viral production systems [12]–[14] using MLV have been developed.

When murine-based packaging cell lines derived from NIH/3T3 were used for retroviral production, the efficiency of the viral vector transductions was inhibited by the proteoglycans secreted by these lines, including parental NIH/3T3 cells [15]. The amphotropic envelope from these packaging lines also contained some materials that inhibit viral infection [16]. To overcome these problems, a human-derived packaging cell line that produces high titer viral supernatant was developed [17]. Purification of retroviral vector was also attempted using a low-speed centrifugation procedure to remove undesirable substances in the viral supernatant and concentrate the retrovirus vector [18], [19]. To increase the chance of contact between the viral vector and target cells, a flow-through transduction method involving the convective flow of retroviral particles through the target cell monolayer was also proposed [20].

Alternatively, we and others have demonstrated that RN is an efficient tool for enhancing gene transfer into hematopoietic stem cells [5]–[7] and T lymphocytes [8], [9] using a retroviral vector system. RN consists of three functional regions: the cell-binding domain (C-domain), the heparin-binding domain (H-domain), and the CS-1 sequence. The C-domain and CS-1 sequence interact with target cells through the integrin receptors VLA-5 and VLA-4 respectively, and the H-domain (which is composed of type III repeats III 12, III l3, and III l4) has the ability to adsorb retroviral virions [21]. Thus, retrovirus-mediated gene transfer is enhanced by co-locating target cells and virions on the RN molecules [5]; because RN's H-domain can bind retrovirus, preloading the retroviral supernatant on an RN-coated vessel will allow transferable inhibitors from the producing cell line to be washed out (RN-bound virus; RBV transduction method). In contrast, gene transfer efficiency does not increase under passive and static preloading conditions, even if the amount of vector used exceeds 0.125 ml/cm^2^ [22]. Viral vector particles cannot be adsorbed under passive conditions, even if the substratum is coated with RN, as these particles are located far from the surface of the substratum.

To utilize the retroviral vector efficiently, active adsorption of the vector is required. To achieve this adsorption, preloading of the vector into an RN-coated plate in combination with low-speed centrifugation or spin transduction (centrifuge cells and vectors together in RN-coated vessel) is sometimes proposed [22]–[24]. However, there are scaling limitations with this preloading method, and it can be difficult to establish the closed system in combination with centrifugation. For procedures such as adoptive T cell transfer gene therapy, large-scale T cell manufacturing is required; approximately 10^8^ cells are needed to manipulate by retroviral vector. In this study, to address these challenges, we utilized RN-coated plastic bag preloading with low-temperature shaking (LTS) and performed large-scale T cell manufacturing of both endoribonuclease MazF-modified CD4^+^ T cells for HIV-1 gene therapy and MAGE-A4 TCR-modified T cells for MAGE-A4-antigen-expressing cancer gene therapy.

## Materials and Methods

### Retroviral vectors and viral production

A fluorescent protein expressing retroviral vector DON-AI-ZsGreen1 was constructed by introducing the ZsGreen1 gene (Clontech, Mountain View, CA) into the multiple cloning site of the pDON-AI-2 plasmid (Takara Bio).

The endoribonuclease MazF-expressing retroviral vector MT-MFR3 is a clinical version of gamma retroviral vector MT-MFR-PL2, which has been described previously [25]–[27]. The MazF transcription cassette of the MT-MFR3 vector is driven by the HIV-1 long terminal repeat (LTR) promoter, and it ends with an HSV-derived polyadenylation signal. An HIV-1-LTR-MazF-polyA cassette was introduced into the multiple cloning site of the pMT plasmid [28] in the opposite direction of the MLV-LTR.

For MAGE-A4 TCR gene therapy, to down-regulate the expression of endogenous TCR by siRNA and thereby enable higher expression of the transgene and reduce the risk of TCR mispairing, an siRNA co-expressing TCR vector, MS-MA24-siTCR, was designed [29].

DON-AI-ZsGreen1, MT-MFR3, and MS-MA24-siTCR vectors were packaged into a PG13 packaging cell line (ATCC CRL-10686). PG13 is a retrovirus packaging cell line derived from mouse fibroblast NIH/3T3 cells by transfection with MLV gag-pol and gibbon ape leukemia virus (GaLV) env plasmids [11]. One advantage of using GaLV pseudotyped retroviruses is that they infect cells from higher mammals more effectively than amphotropic murine retroviruses. The GaLV receptor has been identified as a high affinity sodium-dependent phosphate transporter present in the cells of many species, except those of mouse origin [30].

The PG13 producer lines were cultured in Dulbecco's modified Eagle's medium (Sigma-Aldrich, St. Louis, MO) supplemented with 10% fetal bovine serum (Life Technologies, Carlsbad, CA). Upon production, this medium was replaced by serum-free GT-T-RetroIII medium (Takara Bio), and the supernatants were harvested as viral vectors and frozen at −80°C until use. Viral RNA titers were determined by directly measuring the RNA copies in the supernatants using the qPCR method using the Retrovirus Titer Set (Takara Bio). Biological titers (infection-forming units [IFU]/ml) were determined by transducing into SUP-T1 cells and measuring the proviral copy number using the qPCR method described below. The biological titer was calculated using the following formula,

[IFU/ml)]  =  [proviral copy number]×[virus dilution rate]×[cell number] / [virus volume].

The MT-MFR3 and MS-MA24-siTCR vectors were manufactured at the Takara Bio Center for Cell and Gene Therapy Facility in accordance with the Japanese Ministry of Health, Labour and Welfare's standards for manufacturing control and quality control of investigational products, i.e., the good manufacturing practice (GMP) for investigational products. The vectors were tested in the following items: stability, sterility, mycoplasma, adventitious viruses in vivo, adventitious viruses in vitro, replication competent retrovirus, endotoxin, bovine adventitious viruses in vitro, vector sequence, and vector titer.

### Cell culture

The Human T lymphoblast cell line SUP-T1 (ATCC CRL-1942) was cultured in RPMI-1640 medium (Sigma-Aldrich) supplemented with 10% fetal bovine serum at 37°C in a 5% CO_2_ incubator.

A gene transfer study of healthy donors' blood samples for the purpose of developing gene therapy research was approved by Takara Bio Inc. 's ethics committee. Peripheral blood samples were obtained from healthy volunteers who gave their written informed consent. Peripheral blood samples were collected through leukapheresis and washed using Cytomate (Baxter Healthcare, Deerfield, IL). Mononuclear cells were isolated using Ficoll-Paque PLUS (GE Healthcare, Castle Hill, Australia) density gradient centrifugation and frozen in liquid nitrogen until use.

Human primary CD4^+^ T cells were cultured in X-VIVO15 (with Gentamycin and Phenol red; Lonza, Walkersville, MD) supplemented with 2 mM of GlutaMAX (Life Technologies), 20 mM of HEPES (Lonza), 1 mM of Sodium Pyruvate (Lonza), 1% minimal essential vitamin mix (MEM Vitamin, Lonza), 100 U/ml of Interleukin (IL)-2 (Proleukin; Novartis, Basel, Switzerland), 0.5% human serum albumin (HSA, CSL Behring GmbH, Marburg, Germany), 5% Human AB Serum (Lonza), and 10 mM of N-Acetylcysteine (Showa Yakuhin Kako, Tokyo, Japan).

Human peripheral blood mononuclear cells (PBMCs) were cultured in GT-T551 medium (Takara Bio) supplemented with 600 U/ml of IL-2, 2.5 µg/ml of Fungizone® (Bristol-Myers Squibb, New York, NY), 60 µg/ml of Streptomycin sulfate (Meiji Seika Pharma, Tokyo, Japan), and 0.6% autologous plasma.

### Retroviral transduction

We sought to develop a novel methodology using RBV transduction, and to explore the optimal methodology of active adsorption comprehensively in small scale, we used RN-coated 24-well plates in the first step. RN was diluted to a concentration of 20 µg/ml with acid citrate dextrose formula A (ACD-A) solution (Terumo, Tokyo, Japan). Each non-tissue-culture-treated 24-well plate was coated by adding 0.5 ml of RN solution to each well and kept at 4°C overnight. To preload the vector, the RN solution was removed, the plates were rinsed once with 1 ml of ACD-A solution, and 1 ml of MT-MFR3 vector [(1) 3.4×10^9^ RNA copies/ml, 3.8×10^9^ RNA copies/ml, (*3*) 6.8×10^9^ RNA copies/ml, (4) 2.4×10^9^ RNA copies/ml] was preloaded into each well of the RN-coated 24-well plates. In the static preloading condition (RBV-Static), the plates were incubated for 3 h at 25°C. In the preloading condition involving the centrifuge (RBV-Spin), the plates were centrifuged at 2,000×g for 2 h at 32°C. To explore another active preloading condition, some plates were incubated at 4°C on a reciprocating shaker at 100 rounds per minute (rpm); this was the RBV-LTS (RetroNectin-bound virus with low-temperature shaking) condition. RBV-LTS experiments were performed four times at time interval settings of (1) 16 to 48 h, (2) 12 to 24 h, (3) 12 to 72 h, and (4) 8 to 20 h. After the preloading, supernatants were removed and each well was washed once with 500 µl of PBS containing 1.5% HSA. One milliliter of SUP-T1 cell suspension was added to each well at a concentration of 5×10^5^ cells/ml immediately after the wash and then incubated at 37°C in 5% CO_2_. On the next day, SUP-T1 cells were diluted and then incubated for an additional 3 days. After the incubation, SUP-T1 cells were collected, genomic DNA was extracted, and gene transfer efficiency was determined using the quantitative PCR (qPCR) method to measure the proviral copy number of the transduced cells, as described below.

### Scaled-up transduction using the RBV-LTS method

To explore the possibility of up-scaling in a closed system, a PermaLife PL325 bag (OriGen Biomedical, Austin, TX) was tested. The PL325 bag has a large surface area of 725 cm^2^, including the top and bottom surfaces of the bag. The RN was diluted to a concentration of 20 µg/ml with ACD-A solution. Each PL325 bag was filled with 60 ml of RN solution and air was removed, after which the bag was kept at 4°C overnight. To preload the vector, the RN solution was removed, the bag was rinsed once with 100 ml of ACD-A solution, and then 180 ml of MT-MFR3 vector (1.4 or 2.9×10^5^ IFU/ml) was preloaded into the bag. After air was removed from the bag, it was incubated at 4°C on a reciprocating shaker at 50 rpm. The speed of the shaker was reduced because of differences in stroke between the 24-well plate and PL325 bag.

The optimal preloading period in the PL325 bag was examined at time intervals of 8 to 48 h. After the preloading, the unbound viral supernatant was discarded, the bag was rinsed once with 100 ml of saline containing 1.5% human serum albumin, and the SUP-T1 cells were added to the bag at a concentration of 5×10^5^ cells/ml (180 ml, a total 9×10^7^ cells) and then incubated at 37°C in 5% CO_2_. One of the advantages of bag transduction is that transduction efficiency can be increased by simply flipping the bag over, as retroviral vector can be adsorbed onto the bag's top and bottom surfaces; thus, for this experiment, the bag was flipped over 2 h later and further incubated. For comparison, transduction into SUP-T1 cells in an RN-coated 24-well plate under RBV-LTS, RBV-Spin, and RBV-Static conditions was also performed in parallel. After the incubation, gene transfer efficiency was determined using the qPCR method to measure the proviral copy number of the transduced cells, as described below.

Next, the optimal time to flip the bag was examined. After preloading using RBV-LTS for 16 h, the viral supernatant was discarded, the bag was rinsed, and the SUP-T1 cells were added and then incubated at 37°C in 5% CO_2_. The optimal time to flip the bag was examined at time intervals of 1 to 8 h. On the next day, the SUP-T1 cells were diluted and then incubated for an additional 3 days. After the incubation, the SUP-T1 cells were collected, genomic DNA was extracted, and gene transfer efficiency was determined using the qPCR method to measure the proviral copy number of the transduced cells, as described below.

### Validation runs for manufacturing endoribonuclease MazF-modified human CD4^+^ T cells

Frozen stocks of PBMCs (5×10^7^ cells/vial) were thawed at 37°C. The cells were immediately transferred into 13 ml of X-VIVO15 culture medium containing 13 µl of 10 mg/ml DNase. After centrifugation (500× g, 5 min), the cells were re-suspended in X-VIVO15 culture medium. To remove the monocytes, the suspended cells were seeded onto a tissue-culture-treated T225 flask at a concentration of 4×10^5^ cells/cm^2^ and incubated for 1 h at 37°C in 5% CO_2_. Thereafter, non-adherent cells were harvested and suspended in PBS containing 0.1% BSA and 2 mM of EDTA. CD8^+^ T cells were then removed from the cell suspension using DynaBeads CD8 (Life Technologies) according to the manufacturer's instructions.

The CD8-depleted (CD4^+^-enriched) cells were re-suspended at a concentration of 5×10^5^ cells/ml in X-VIVO15 culture medium and seeded into a PL325 bag (300 ml/bag). Anti-CD3 and anti-CD28 monoclonal antibodies conjugated beads (CD3/CD28 beads, Dynabeads® Human T-Expander CD3/CD28, Life Technologies) were then added to the cell culture at a cell-to-bead ratio of 1:3. The cells were then incubated at 37°C in 5% CO_2_ (on day 0).

On day 2, 180 ml of the MazF vector MT-MFR3 (5.7×10^5^ IFU/ml) was preloaded into an RN-coated PL325 bag and then incubated at 4°C on a reciprocating shaker at 50 rpm for 16 h. On day 3, the unbound viral supernatant was discarded, the bag was rinsed once with 100 ml of saline containing 1.5% human serum albumin, and activated CD4^+^ T cells were added to the bag at a concentration of 5×10^5^ cells/ml (180 ml, a total of 9×10^7^ cells) and incubated at 37°C in 5% CO_2_. After 1 h of incubation, the bag was flipped over and further incubated overnight. On day 4, the transduction was repeated using the same procedure. On day 5, the cells were transferred to a CultiLife Eva culture bag (one surface area is 640 cm^2^, Takara Bio), diluted as necessary, and cultured until day 11.

### Validation runs for manufacturing TCR-modified human T cells

Frozen stocks of PBMCs were thawed at 37°C. The cells were immediately transferred into 13 ml of GT-T551 containing 13 µl of 10 mg/ml DNase. After centrifugation (500×g, 5 min), the cells were re-suspended in GT-T551 culture medium. The cells were incubated at 37°C in 5% CO_2_ in an RN- and anti-CD3 (Orthoclone OKT3; Janssen pharmaceutical, Titusville, NJ)-coated CultiLife 215 bag (Takara Bio). In brief, the CultiLife 215 bag was pre-coated with 25 µg/ml of RN and 5 µg/ml of OKT3 at 37°C for 5-10 h (25 ml/bag). After the coating, the bag was rinsed three times with culture medium, and cells were seeded at a concentration of 2×10^5^ cell/ml (1.4×10^5^ cells/cm^2^).

On day 3, an RN-coated PL325 bag was preloaded with 180 ml of the TCR vector MS-MA24-siTCR (8.7×10^9^ RNA copies/ml) and then incubated at 4°C on a reciprocating shaker at 50 rpm for 16 h. On day 4, the unbound viral supernatant was discarded, the bag was rinsed once with 100 ml of saline containing 1.5% human serum albumin, activated PBMCs were added to the bag at a concentration of 4×10^5^ cells/ml (160 ml, a total 6.4×10^7^ cells), and the bag was incubated at 37°C in 5% CO_2_. After 1 h of incubation, the bag was flipped over and further incubated overnight. On day 5, the cells were transduced again for 1 h and incubated for an additional 4 h after flipping the bag. They were then transferred to a CultiLife Eva culture bag, diluted as necessary, and expanded until day 10.

### qPCR method

Retroviral gene transduction efficiency was analyzed by measuring the proviral DNA copy number of the transduced cells. More than four days after transduction, transduced cells were harvested and genomic DNA was extracted. As a negative control, non gene-modified cells were cultured in parallel with gene-modified cells and DNA was extracted. The concentration and purity of the extracted DNA were analyzed using the NanoDrop Spectrophotometer ND-1000 (Thermo Fisher Scientific, Boston, MA). The proviral copy number of the retrovirus was measured using the qPCR method. A set of specific primers and probes (Provirus Copy Number Detection Primer Set, Human, Takara Bio) was used to detect either the packaging signal region of retrovirus or the human interferon-gamma (IFNγ) gene to determine the ratio between the copy numbers of these genes. Quantification of the provirus gene and the human IFNγ gene was performed through two separate reactions using the same amount of extracted genomic DNA.

To analyze vector adsorption efficiency across different RBV methods, RNAs were directly extracted from vector preloaded vessels as follows: MT-MFR3 (2.9×10^5^ IFU/ml) vector was preloaded into 24-well RN-coated plates and subjected to the RBV-Static, -Spin, or -LTS methods (0.5 ml/cm^2^). Scaled-up preloading was also performed using RN-coated PL325 bags that were subjected to the RBV-LTS method (0.5 ml/cm^2^). After the preloading, the vessels were washed once with saline containing 1.5% human serum albumin, and retroviral RNAs were directly extracted using extraction solvent (RNAiso Plus, Takara Bio). As a control, retroviral RNAs were extracted directly from the same amount of input retrovirus supernatant. The extracted RNA was subjected to qPCR using the Retrovirus Titer Set (Takara Bio) and One Step SYBR PrimeScript RT-PCR Kit (Takara Bio). The Retrovirus Titer Set is designed to quantify viral RNA. Using this kit with the One Step SYBR PrimeScript RT-PCR Kit, we can specifically and quantitatively detect RNAs of MLV-based retroviral vectors. The standard curve was prepared using 10 µl of serially diluted RNA control template (10^2^ to 10^7^ copies/µl). Adsorption efficiencies were calculated by dividing the number of recovered RNA copies by the total number of input RNA copies.

The proviral copy number of gene-modified cells and the retroviral RNA copy number were measured in duplicate using the Thermal Cycler Dice Real Time System TP-800 (Takara Bio). Data analyses were performed using Multiplate RQ software (Takara Bio), and the analyzed data were processed using Microsoft Excel 2007 software.

### Flow cytometry analysis

Flow cytometry was used to analyze surface phenotype and transduction efficiency. The following monoclonal antibodies that detect surface proteins were used: PerCP-conjugated anti-CD3 antibody (BD Biosciences, San Jose, CA), APCcy7-conjugated anti-CD4 (BD Biosciences), FITC-or APCcy7-conjugated anti-CD8 (BD Biosciences), RD1-conjugated anti-CD45RA (Beckman Coulter, Fullerton, CA), and FITC- or APC-conjugated CCR7 (R&D Systems, Minneapolis, MN). To analyze the gene transfer efficiency of TCR-modified T cells, we double-stained the transduced PBMCs with FITC-conjugated anti-CD8 and PE-conjugated MAGE-A4_143-151_/HLA-A*2402 tetramers (Ludwig Institute for Cancer Research, New York, NY). Samples were run through a FACS CantoII flow cytometer (BD Biosciences). Data were analyzed using FACS Diva software (BD Biosciences).

### Data analysis

Data were analyzed for their statistical significance by the Student t-test.

## Results

### Comparison of preloading method

At first, we evaluated the RBV-LTS preloading temperature on a small scale using 24-well plates. DON-AI-ZsGreen1 vector was preloaded into each well of the RN-coated 24-well plates. When the plates were incubated for 24 h at 4°C, 16°C, or 37°C using a rocking shaker (RBV-LTS) at 35 rpm, gene transfer efficiencies (ZsGreen1-positive percentage) were 13.4, 12.4, and 0.25%, respectively. Preloading below 16°C was better for RBV-LTS method and we chose 4°C for the following experiments.

**Figure 1:**
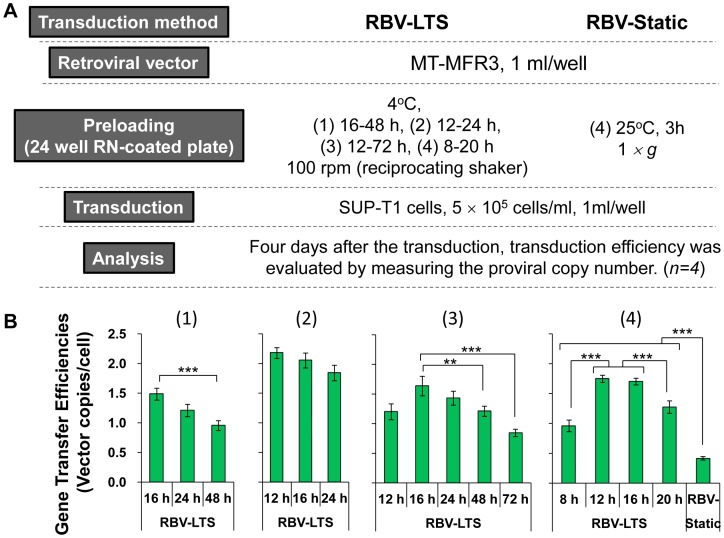
Comparison of preloading methods for retroviral gene transfer assisted by RN. (A) Outline of the experiment. MT-MFR3 vector was preloaded into each well of an RN-coated 24-well plate. The plates were incubated at 4°C on a reciprocating shaker at 100 rpm for (1) 16 to 48 h, (2) 12 to 24 h, (3) 12 to 72 h, and (4) 8 to 20 h (RBV-LTS). For comparison, the plate was incubated at 25°C for 3 h (RBV-Static) (4). After the preloading, SUP-T1 cells were transduced via each method. (B) Retroviral gene transfer efficiencies into SUP-T1 cells under RBV-LTS and RBV-Static conditions. After the transduction, SUP-T1 cells were collected, genomic DNA was extracted, and gene transfer efficiency was determined using the qPCR method by measuring the proviral copy number of the transduced cells. All data represent mean ± SD. Statistical analysis was performed by Student *t*-test (^**^ *p*<0.01, ^***^ *p*<0.001). RN, RetroNectin; RBV, RN-bound virus; LTS, low-temperature shaking; IFU, infection-forming units.

Next, endoribonuclease MazF-expressing MT-MFR3 vector was preloaded into each well of the RN-coated 24-well plates. As the MazF-expressing retroviral vector had no marker gene and expression of MazF was designed to be induced only upon HIV-1 replication, measurement of proviral copy number was the only method to evaluate the transduction efficiency of this vector. Given the half-life of retroviral vector within the cell is between 5.5 and 7.5 h [31] and unintegrated DNA in cytoplasm might be digested by nucleases [32], the analyses performed four days after transduction avoided the possible amplification of episomal unintegrated retroviral DNA. The experiments were performed four times using various time interval settings as described in Materials and Methods. It reduced the transduction efficiency significantly when vector was preloaded with RBV-LTS method over 48 h [Figure 1B (1), (3)]. When the plates were incubated for 8 to 20 h at 4°C using a reciprocating shaker (RBV-LTS) at 100 rpm, the transduced vector copy number significantly increased compared to the results obtained using the conventional RBV-Static preloading method [Figure 1B (4)]. The optimal incubation period was 12 to 16 h when an RN-coated 24-well plate was used. The active adsorption achieved through the movement of vector supernatant at the time of preloading resulted in increased transduction efficiency.

### Scaled-up transduction using the RBV-LTS method

**Figure 2:**
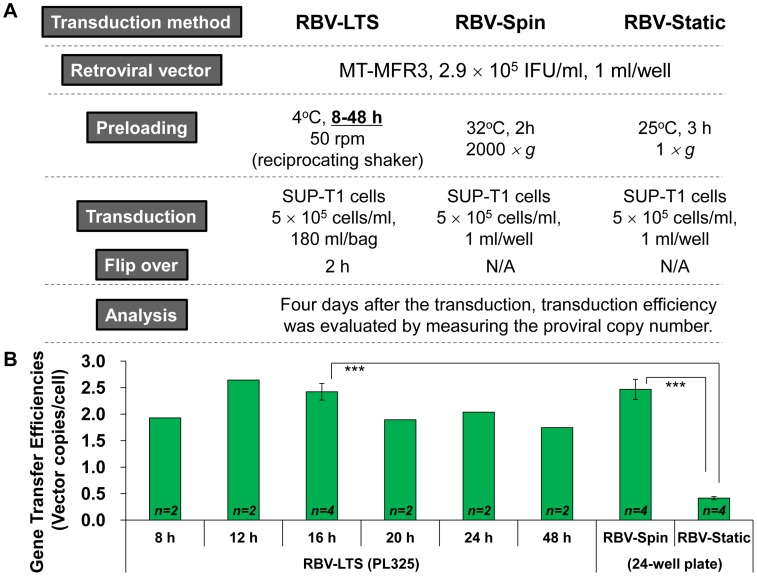
Optimal amount of time for preloading using a scaled-up transduction procedure with the RBV-LTS method. (A) Outline of the experiment. MT-MFR3 vector was preloaded into an RN-coated PL325 bag. 180 ml of MT-MFR3 vector was preloaded into the bag, which was then incubated at 4°C on a reciprocating shaker at 50 rpm. The optimal preloading period in the PL325 bag was examined at the time intervals of 8 to 48 h. For comparison, transduction into SUP-T1 cells in RN-coated 24-well plates under RBV-Static and RBV-Spin conditions was also performed in parallel. (B) Retroviral gene transfer efficiencies into SUP-T1 cells under the large-scale RBV-LTS condition. After the transduction, SUP-T1 cells were collected, genomic DNA was extracted, and gene transfer efficiency was determined using the qPCR method. All data represent mean ± SD. Statistical analysis was performed by Student *t*-test (^***^ *p*<0.001). N/A, not applicable.

To explore the possibility of up-scaling transduction within a closed system, PL325 bags were coated with RN and transduction was performed using the RBV-LTS method. At first, to determine the optimal incubation time for preloading in a closed system, MT-MFR3 retroviral vector was preloaded into RN-coated PL325 bags, the bags were incubated at 4°C on a reciprocating shaker at 50 rpm and SUP-T1 cells were transduced. For comparison, MT-MFR3 retroviral vector was also preloaded into RN-coated 24-well plates, in which SUP-T1 cells were transduced in RBV-Spin and RBV-Static conditions. As Figure 2B shows, bag preloading for 12 or 16 h using RBV-LTS resulted in transduction efficiency that was equal to that of the RBV-Spin method and increased more than fivefold compared to the conventional RBV-Static preloading method. Thus, by using the RN-coated bag, the transduction process was efficiently and easily scaled up.

**Figure 3:**
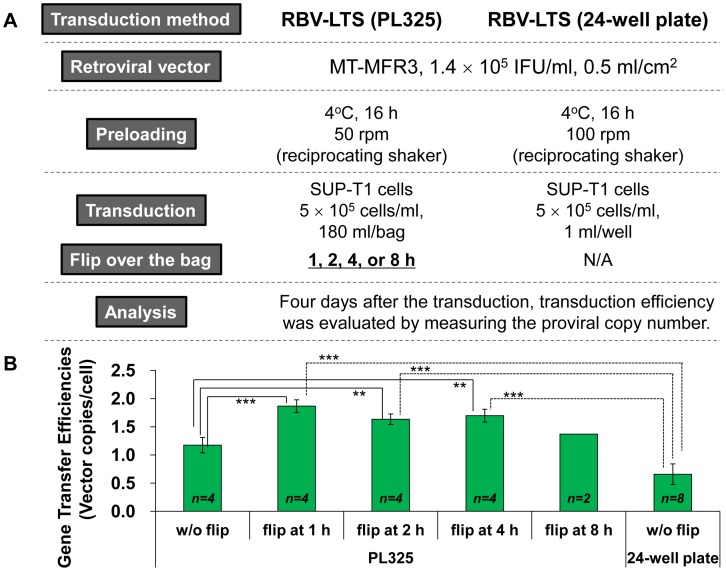
The optimal time for flipping over the bag in a scaled-up transduction procedure using the RBV-LTS method. (A) Outline of the experiment. MT-MFR3 vector was preloaded into an RN-coated PL325 bag. The bag was incubated at 4°C on a reciprocating shaker at 50 rpm for 16 h. After preloading, the bag was rinsed once, SUP-T1 cells were added to the bag, and the optimal time to flip the bag over was examined at time intervals of 1 to 8 h. For comparison, transduction into SUP-T1 cells in an RN-coated 24-well plate under RBV-LTS was also performed in parallel. (B) Retroviral gene transfer efficiencies into SUP-T1 cells under the large-scale RBV-LTS condition. After transduction, SUP-T1 cells were collected, genomic DNA was extracted, and gene transfer efficiency was determined using the qPCR method. All data represent mean ± SD. Statistical analysis was performed by Student *t*-test (^**^ *p*<0.01, ^***^ *p*<0.001). w/o, without.

Because bag transduction has the advantage of allowing for adsorption of retroviral vector onto the top and bottom surfaces of the bag, the optimal time for flipping the bag was examined after the inoculation of the SUP-T1 cells. As shown in Figure 3B, transduction efficiency increased with statistical significance when bags were flipped between 1 to 4 hours after transduction. When the bag was flipped 1 h after transduction, the transduced vector copy number increased 1.6-fold. These data suggest that simply flipping the bag facilitated more efficient utilization of the retroviral vector, as it allowed the vector to be adsorbed onto both sides of PL325 bag.

### Quantification of bound virus harvested from the RN-coated substratum

To confirm that the enhanced gene transfer was the result of more efficient viral adsorption, we measured the attached retroviral vector directly using the qPCR method. Adsorption efficiencies were calculated by dividing the number of recovered RNA copies by the total number of input RNA copies. As Table 1 shows, in the absence of RN, we were not able to observe effective adsorption of retrovirus vector to the PL325 as well as 24-well plate. When the RN-coated PL325 bag was used with the RBV-LTS, retroviral vectors were adsorbed with an equivalent efficiency compared to RBV-Spin method and more than eightfold higher compared to the RBV-Static method (*p*<0.01). The adsorption efficiency correlates with the transduction efficiency shown in Figure 2.

**Table 1:** Retroviral vector adsorption efficiency using the RBV-LTS method.

Vessel	Method	RN coating	Adsorption efficiency (%)
24-well plate	Static (32°C, 4 h)	−	<0.2
24-well plate	Static (32°C, 4 h)	+	4.3±1.4
24-well plate	Spin (2000× *g*, 32°C, 2 h)	−	5.0±0.7
24-well plate	Spin (2000× *g*, 32°C, 2 h)	+	40.8±2.9^***^
24-well plate	LTS (100 rpm, 4°C, 16 h)	−	<0.2
24-well plate	LTS (100 rpm, 4°C, 16 h)	+	26.0±8.4^*^
PL325	LTS (50 rpm, 4°C, 16 h)	−	<0.2
PL325	LTS (50 rpm, 4°C, 16 h)	+	36.5±10.2^**^

MT-MFR3 vector was preloaded into RN-coated 24-well plates, which were then subjected to the RBV-static, -Spin, or -LTS methods. Scaled-up preloading was also performed using an RN-coated PL325 bag with the RBV-LTS method. After the preloading, the residual retroviral RNAs were directly extracted using extraction solvent. The amount of retroviral RNA present was quantified using the qPCR method. Adsorption efficiencies were calculated by dividing the number of recovered RNA copies by the total number of input RNA copies. Experiments were conducted in triplicate, and all data represent mean ± SD. Statistical analysis was performed by Student *t*-test between active adsorptions (Spin or LTS) and passive adsorption (Static). (^*^ *p*<0.05, ^**^ *p*<0.01, ^***^ *p*<0.001).

### Validation runs for manufacturing endoribonuclease MazF-modified human CD4^+^ T cells

**Figure 4:**
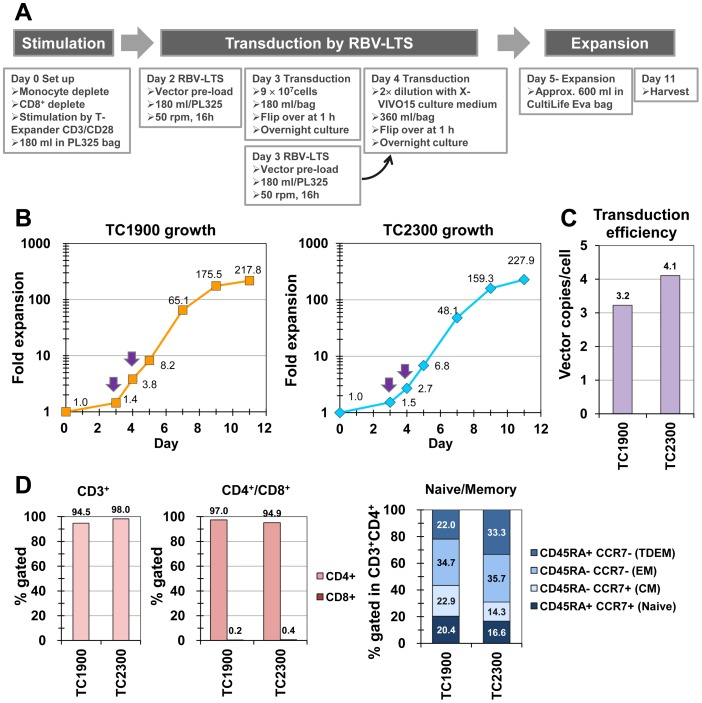
Validation runs for manufacturing endoribonuclease MazF-modified human CD4^+^ T cells. (A) Schematic diagram of the experiment. Primary human CD4^+^ T cells were stimulated, transduced with MT-MFR3 vector using the RBV-LTS method, and further expanded. (B) Growth curve of two validation runs of healthy donors' CD4^+^ T cells. Arrows indicate the time of the transduction. (C) Retroviral gene transfer efficiencies into CD4^+^ T cells under the large-scale RBV-LTS condition. Gene transfer efficiency was determined using the qPCR method to determine the proviral copy numbers (D) The percentage of CD3 positive cells among the expanded cells was measured by gating the live cells based on the FSC/SSC parameters during the flow cytometry analysis (left panel). After gating for CD3, cells were analyzed for CD4^+^ or CD8^+^ expression (middle panel). CD3^+^ CD4^+^ cells were also analyzed for CD45RA and CCR7 expression using flow cytometry (right panel). CM, central memory; EM, effector memory; TDEM, terminally differentiated effector memory.

MazF-modified CD4^+^ T cells were manufactured from the primary CD4^+^ T cells of two healthy donors (TC1900 and TC2300) by transducing with the MazF endoribonuclease-expressing retroviral vector MT-MFR3. An outline of MazF-modified CD4^+^ T cell manufacturing procedure is depicted in Figure 4A. The CD4^+^ T cells expanded over 200-fold during the 11 days of culture, yielding over 98% viable cells (Figure 4B). The gene transfer efficiencies for TC1900 and TC2300 were 3.2 and 4.1 copies/cell, respectively (Figure 4C). The gene transfer efficiencies of single round of transduction on day 3 for TC1900 and TC2300 were 1.5 and 1.8 copies/cell respectively, which were just half of the efficiencies described above. In addition, if we use the same amount of vector, gene transfer efficiencies of repeating the transduction by diluting twofold was 3.4 copies/cell for TC2300, which was significantly higher than that of single round of transduction by non-diluted vector (1.8 copies/cell). Thus, repeating the transduction was more effective to enhance the transduction efficiencies.

The majority of expanded cells were CD3^+^ CD4^+^ T cells, with a variety of cell populations present including CD45RA^+^ CCR7^+^ naïve phenotype T cells, CD45RA^−^ CCR7^+^ central memory phenotype T cells (CM), CD45RA^−^ CCR7^−^ effector memory phenotype T cells (EM), and CD45RA^+^ CCR7^−^ terminally differentiated effector memory phenotype T cells (TDEM) (Figure 4D). The gene transduction efficiency, viabilities and surface antigen markers are satisfactory when the MazF endoribonuclease-modified T cell is used for HIV-1 gene therapy.

### Validation runs for manufacturing TCR-modified human T cells

**Figure 5:**
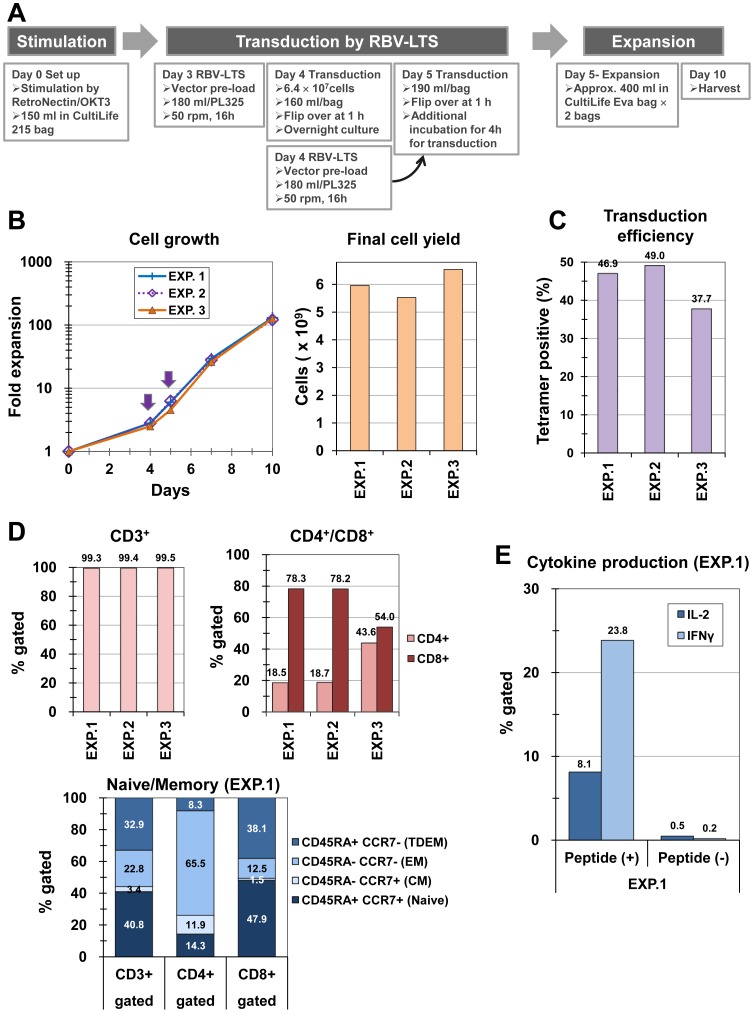
Validation runs for manufacturing TCR-modified human T cells. (A) Schematic diagram of the experiment. Primary human PBMCs were stimulated, transduced with MS-MA24-siTCR vector using the RBV-LTS method, and further expanded. (B) Growth curve of three validation runs of healthy donors' PBMCs. EXP. 1 and 2 are the results from Donor TC2900 and EXP 3 is from Donor TC1900. Arrows indicate the time of the transduction. (C) Retroviral gene transfer efficiencies into PBMCs under the large-scale RBV-LTS condition. Gene transfer efficiency was determined based on the percentages of tetramer-positive cells among the CD8^+^ cells. (D) The percentage of CD3 positive cells among the expanded cells was measured by gating the live cells based on the FSC/SSC parameters during the flow cytometry analysis (upper left panel). After gating for CD3, cells were analyzed for CD4^+^ or CD8^+^ expression (upper right panel). CD3^+^ cells were also analyzed for CD45RA and CCR7 expression using flow cytometry (bottom panel). (E) Antigen specific IL-2 and IFNγ production achieved by stimulating the gene-modified cells with MAGE-A4 peptide-pulsed T2A24 cells. IL-2, interleukin-2; IFNγ, interferon-gamma; Approx., approximately; EXP., Experiment.

TCR-modified T cells were manufactured from the primary PBMCs of two healthy donors (TC1900 and TC2900) by transducing with the TCR-expressing retroviral vector MS-MA24-siTCR. An outline of the TCR-modified T cell manufacturing procedure is depicted in Figure 5A. We used TC2900 PBMCs for Experiments 1 and 2 and TC1900 for Experiment 3. The PBMCs expanded over 100-fold (an average of 126±4.8-fold) during 10 days of culture, yielding over 98% viable cells (Figure 5B). The gene transfer efficiencies were determined by detecting the expression of TCR by staining the transduced cells with tetramer. The gene transfer efficiencies of the three independent manufacturing runs were 46.9%, 49.0%, and 37.7%, measured based on the percentages of tetramer-positive cells among the CD8^+^ cells (Figure 5C). The gene transfer efficiencies based on the proviral copy number of the three independent manufacturing runs were 2.4, 2.5, and 1.5 copies/cell, respectively.

The majority of expanded cells were CD3^+^ T cells, with 78% CD8^+^ and 19% CD4^+^ for TC2900 (Experiment 1 and 2) and 54% CD8^+^ and 44% CD4^+^ for TC1900 (Experiment 3) (Figure 5D). Naïve/memory phenotype populations were similar among the three experiments, and one representative result is shown in Figure 5D. When cells were gated in CD3^+^ populations, the majority were CD45RA^+^ CCR7^+^ naïve phenotype T cells. A similar tendency was observed when cells were gated in CD8^+^ populations. When cells were gated in CD4^+^ populations, the majority were effector memory phenotype T cells (Figure 5D). Antigen-specific cytokine production (IL-2 and interferon-gamma) was also observed when stimulating the gene-modified cells with MAGE-A4 peptide-pulsed T2A24 cells (Figure 5E). The gene transduction efficiency, viabilities, surface antigen markers, and potency of gene-modified cells are satisfactory when the TCR-modified T cell is used for adoptive transfer gene therapy.

## Discussion

Recently, gene therapy became one of the most promising approaches to curing genetic diseases [33], [34], cancers [35]–[37], and infectious diseases [38] that are resistant to conventional treatments. Many types of protocols have been developed so far, including viral vector approaches. Among these, ex vivo gene therapy by editing cells with viral vectors to render them resistant to disease is the most widely used approach to treating patients and many protocols have been developed. In particular, adoptive transfer therapies with genetically modified T cells appear to be clinically valuable vehicles for gene therapy targeting cancers [39]–[42] and HIV infections [43]–[46]. Lentiviral vector, retroviral vector, or adenoviral vector are used in these trials. Gamma-retroviral vector has an advantage of its easier manufacturing in large scale. Numerous methodologies for improving retroviral transduction efficiency have been tested, and a fragment of fibronectin named RetroNectin (RN) was found to increase the efficiency of retroviral transduction [5]–[9].

Despite such successes, the clinical application of retrovirus vector-mediated gene transfer has sometimes been hampered by low gene transfer efficiencies. The factors contributing to low gene transfer efficiency include the presence of molecules inhibiting transduction in the retroviral vector preparations or a low viral titer. To overcome these problems, an improved transduction procedure had been proposed, which entails preloading the viral vector onto RN [6], [7]. Preloading the retroviral vector onto an RN-coated substratum (RN-bound virus infection method; RBV) can remove the inhibitory molecules efficiently, thereby increasing the transduction efficiency so that it is directly proportional to the viral titer. Moreover, the RBV transduction method in combination with low-speed centrifugation (RBV-Spin) improves transduction efficiency dramatically because it facilitates more efficient adsorption of viral vector onto the RN-coated substratum [21]. However, due to the capacity of the centrifuge, the RBV-Spin method is limited in terms of the number of cells that can be processed. In addition, it can be difficult to apply closed-system manufacturing using RBV-Spin because only certain types of vessels can be used. To treat patients using a T cell-based gene therapy approach, it is necessary to process a large number of gene-modified T cells (10^9^–10^10^ per dose); thus, we chose to use a plastic bag (725 cm^2^) for transduction. Our study demonstrates a novel protocol for preloading the retroviral vector into a plastic bag and allowing gene transfer to occur on a large scale in an appropriate T cell growth medium in the absence of inhibitory molecules.

We initially explored the possibility of enhancing retroviral vector adsorption on an RN-coated substratum by shaking the vessel using an RN-coated 24-well plate. We used the GMP-grade retroviral vector MT-MFR3, which expresses endoribonuclease MazF (used for HIV-1 gene therapy) [25]–[27]. As this vector has no marker gene, gene transfer efficiencies were evaluated by measuring the proviral copy numbers of the transduced cells. We found that preloading with shaking overnight below 16°C is better than preloading at 37°C and we chose 4°C for our experiments. A speed of 100 rpm is optimal when using a reciprocating shaker for a 24-well plate (data not shown). Preloading for 12 to 16 h yielded a fourfold increase in gene transfer efficiency compared to the static preloading method [Figure 1B (4)]. Thus, we found that the RBV transduction method, in combination with low-temperature shaking (RBV-LTS), has great potential to improve gene transfer efficiency.

Next, we used an RN-coated gas-permeable plastic bag to examine the use of RBV-LTS with gene transduction on a larger scale. Efficient gene transfer will only occur if the RN is coated on certain materials; for example, ethylene vinyl acetate and polyolefin bags do not work for RN-coating and transduction, while polystyrene, cyclo-olefin, polyethylene, and Teflon bags work well (data not shown). We chose the PermaLife bag PL325, which is made from Teflon film, for our scaled up experiments. After the RN-coated PL325 bag was filled with the viral vector, the bag was then shaken at 4°C for 16 h on a reciprocating shaker. Completely removing the remaining air from the bag allowed the viral vector to be adsorbed onto the top and bottom surfaces of the RN-coated PL325 bag, and it was expected that simply flipping the bag over would help increase the gene transfer efficiency. Indeed, when the bag was flipped over after 1 to 4 h preloading, the transduced vector copy number increased with statistical significance (Figure 3). Surprisingly, the gene transfer efficiency of this scaled-up transduction procedure using an RN-coated PL325 bag was identical to that of the RBV-Spin method, which was thought to be the most efficient RN-assisted transduction method but was difficult to scale up. In fact, the retroviral vector adsorption efficiency of the RBV-LTS method was significantly higher than that of the RBV-Static method and equivalent to that of the RBV-Spin method (Table 1).

Finally, we applied this optimized transduction procedure for manufacturing engineered T cells in our original gene therapy programs. At first, CD4^+^ T cells were modified by MT-MFR3 retroviral vector to confer resistance to HIV-1 replication in the cells. To validate the transduction procedure, healthy donors' primary CD4^+^ T cells were used instead of cells from HIV-1 patients. The transductions were performed on day 3 and day 4. As we preloaded the vector and removed the supernatants, the cells were transduced in their own growth medium in the absence of vector supernatants. Thus, the cells were not affected by the culture fluid of retrovirus-producer cells and were easily and efficiently expanded. By using a single PL325 bag for each transduction, we were able to obtain a 10^10^ dose of MazF-modified CD4^+^ T cells. The gene transfer efficiencies, viabilities and the surface antigen markers of the two donors' manufactured CD4^+^ T cells were satisfactory when the MazF endoribonuclease-modified CD4^+^ T cell was used for HIV-1 gene therapy. A phase I clinical trial using this transduction method is underway in the United States (see clinicaltrials.gov, identifier NCT01787994).

Next, validation runs to modify PBMCs using MS-MA24-siTCR vector to re-directing T cells to kill MAGE-A4 antigen-expressing cancers were performed. In this gene therapy program, PBMCs were stimulated in a combination of anti-CD3 antibody (OKT3) and RN. When gene therapy based on PBMCs was examined, the fold expansion of the gene-transduced T cells under stimulation by immobilized RN together with OKT3 enhanced cell proliferation while conserving the naïve phenotype of T cells in comparison to the anti-CD3 only or anti-CD3 and anti-CD28 monoclonal antibody co-stimulation methods [47], [48]. Additionally, RN increased gene transfer efficiency when used in concert with the RBV-LTS method. These features are desirable for T cell gene therapy, as it is necessary to prepare over 5×10^9^ gene-modified cells at a time. We performed three validation runs using two donors' PBMCs and consistently obtained a 5×10^9^ dose of gene-modified T cells by using a single PL325 bag for each transduction. Consequently, the manufactured CD8^+^ T cells preserved the naïve phenotype of the T cell population, suggesting that one can expect to see increased persistence of genetically modified T cells in vivo [48]. The gene transduction efficiency, viabilities, surface antigen markers, and potency of the gene-modified cells are satisfactory when the TCR-modified T cells are used for adoptive transfer gene therapy.

In the present study, we demonstrated the optimized large scale engineered T-cell manufacturing using gamma-retroviral vectors. The gene therapy field would also be interested in the optimization of engineered T-cell manufacturing using HIV-based lentiviral vectors, and we are now addressing this issue.

Commercialization of engineered T cell-based adoptive transfer gene therapies should focus on ensuring consistently efficient transduction in the manufacture of T cells, as such efficiency lowers costs and reduces the risk of contamination. We showed that an RBV-LTS method using a gas-permeable plastic bag (which could adsorb retroviral vector efficiently onto its surface) could efficiently transduce genes into T cells in a large-scale closed system. Closed-system bag transduction is advantageous because it reduces the risk of contamination and allows for production on a larger scale. Clinical scale GMP-transduction of primary human T lymphocytes using RN had previously been reported [49], in which T cells were transduced under static condition in the presence of vector containing supernatants or RBV-Spin method. Our improved method has the benefit that T cells were transduced in their own growth medium and retroviral vectors adsorbed on both top and bottom surface of the bag were efficiently utilized. This technology also reduces consumption of viral vector. In summary, RBV-LTS facilitates higher gene transduction efficiency and can be used for large-scale bag transduction. Moreover, the method is very simple and cost-effective, it can be easily integrated into an automated system for manufacturing gene-modified cells, and it is expected to improve current clinical gene therapy protocols.

## References

[1] Yoder MC, Williams DA (1995) Matrix molecule interactions with hematopoietic stem cells. Exp Hematol 23: 961–967. 7635183

[2] Ruoslahti E (1988) Fibronectin and its receptors. Annu Rev Biochem 57: 375–413. 2972252 10.1146/annurev.bi.57.070188.002111

[3] Hynes RO (1992) Integrins: versatility, modulation, and signaling in cell adhesion. Cell 69: 11–25. 1555235 10.1016/0092-8674(92)90115-s

[4] Kimizuka F, Taguchi Y, Ohdate Y, Kawase Y, Shimojo T, et al (1991) Production and characterization of functional domains of human fibronectin expressed in *Escherichia coli* . J Biochem 110: 284–291. 1761524 10.1093/oxfordjournals.jbchem.a123572

[5] Hanenberg H, Xiao XL, Dilloo D, Hashino K, Kato I, et al (1996) Colocalization of retrovirus and target cells on specific fibronectin fragments increases genetic transduction of mammalian cells. Nat Med 2: 876–882. 8705856 10.1038/nm0896-876

[6] Hanenberg H, Hashino K, Konishi H, Hock RA, Kato I, et al (1997) Optimization of fibronectin-assisted retroviral gene transfer into human CD34+ hematopoietic cells. Hum Gene Ther 8: 2193–2206. 9449373 10.1089/hum.1997.8.18-2193

[7] Chono H, Yoshioka H, Ueno M, Kato I (2001) Removal of inhibitory substances with recombinant fibronectin-CH-296 plates enhances the retroviral transduction efficiency of CD34(+)CD38(-) bone marrow cells. J Biochem 130: 331–334. 11530007 10.1093/oxfordjournals.jbchem.a002990

[8] Pollok KE, Hanenberg H, Noblitt TW, Schroeder WL, Kato I, et al (1998) High-efficiency gene transfer into normal and adenosine deaminase-deficient T lymphocytes is mediated by transduction on recombinant fibronectin fragments. J Virol 72: 4882–4892. 9573255 10.1128/jvi.72.6.4882-4892.1998 PMC110042

[9] Dardalhon V, Noraz N, Pollok K, Rebouissou C, Boyer M, et al (1999) Green fluorescent protein as a selectable marker of fibronectin-facilitated retroviral gene transfer in primary human T lymphocytes. Hum Gene Ther 10: 5–14. 10022526 10.1089/10430349950019147

[10] Markowitz D, Goff S, Bank A (1988) Construction and use of a safe and efficient amphotropic packaging cell line. Virology 167: 400–406. 2462307

[11] Miller AD, Garcia JV, von Suhr N, Lynch CM, Wilson C, et al (1991) Construction and properties of retrovirus packaging cells based on gibbon ape leukemia virus. J Virol 65: 2220–2224. 1850008 10.1128/jvi.65.5.2220-2224.1991 PMC240569

[12] Pear WS, Nolan GP, Scott ML, Baltimore D (1993) Production of high-titer helper-free retroviruses by transient transfection. Proc Natl Acad Sci U S A 90: 8392–8396. 7690960 10.1073/pnas.90.18.8392 PMC47362

[13] Naviaux RK, Costanzi E, Haas M, Verma IM (1996) The pCL vector system: rapid production of helper-free, high-titer, recombinant retroviruses. J Virol 70: 5701–5705. 8764092 10.1128/jvi.70.8.5701-5705.1996 PMC190538

[14] Yang S, Delgado R, King SR, Woffendin C, Barker CS, et al (1999) Generation of retroviral vector for clinical studies using transient transfection. Hum Gene Ther 10: 123–132. 10022537 10.1089/10430349950019255

[15] Le Doux JM, Morgan JR, Snow RG, Yarmush ML (1996) Proteoglycans secreted by packaging cell lines inhibit retrovirus infection. J Virol 70: 6468–6473. 8709284 10.1128/jvi.70.9.6468-6473.1996 PMC190682

[16] Forestell SP, Bohnlein E, Rigg RJ (1995) Retroviral end-point titer is not predictive of gene transfer efficiency: implications for vector production. Gene Ther 2: 723–730. 8750011

[17] Davis JL, Witt RM, Gross PR, Hokanson CA, Jungles S, et al (1997) Retroviral particles produced from a stable human-derived packaging cell line transduce target cells with very high efficiencies. Hum Gene Ther 8: 1459–1467. 9287146 10.1089/hum.1997.8.12-1459

[18] Seppen J, Kimmel RJ, Osborne WR (1997) Serum-free production, concentration and purification of recombinant retroviruses. Biotechniques 23: 788–790. 9383536 10.2144/97235bm04

[19] Bowles NE, Eisensmith RC, Mohuiddin R, Pyron M, Woo SL (1996) A simple and efficient method for the concentration and purification of recombinant retrovirus for increased hepatocyte transduction *in vivo* . Hum Gene Ther 7: 1735–1742. 8886844 10.1089/hum.1996.7.14-1735

[20] Chuck AS, Palsson BO (1996) Consistent and high rates of gene transfer can be obtained using flow-through transduction over a wide range of retroviral titers. Hum Gene Ther 7: 743–750. 8919596 10.1089/hum.1996.7.6-743

[21] Moritz T, Dutt P, Xiao X, Carstanjen D, Vik T, et al (1996) Fibronectin improves transduction of reconstituting hematopoietic stem cells by retroviral vectors: evidence of direct viral binding to chymotryptic carboxy-terminal fragments. Blood 88: 855–862. 8704241

[22] Tonks A, Tonks AJ, Pearn L, Mohamad Z, Burnett AK, et al (2005) Optimized retroviral transduction protocol which preserves the primitive subpopulation of human hematopoietic cells. Biotechnol Prog 21: 953–958. 15932279 10.1021/bp0500314

[23] Zhou P, Lee J, Moore P, Brasky KM (2001) High-efficiency gene transfer into rhesus macaque primary T lymphocytes by combining 32 degrees C centrifugation and CH-296-coated plates: effect of gene transfer protocol on T cell homing receptor expression. Hum Gene Ther 12: 1843–1855. 11589827 10.1089/104303401753153901

[24] Quintas-Cardama A, Yeh RK, Hollyman D, Stefanski J, Taylor C, et al (2007) Multifactorial optimization of gammaretroviral gene transfer into human T lymphocytes for clinical application. Hum Gene Ther 18: 1253–1260. 18052719 10.1089/hum.2007.088

[25] Chono H, Matsumoto K, Tsuda H, Saito N, Lee K, et al (2011) Acquisition of HIV-1 resistance in T lymphocytes using an ACA-specific *E. coli* mRNA interferase. Hum Gene Ther 22: 35–43. 20649483 10.1089/hum.2010.001

[26] Chono H, Saito N, Tsuda H, Shibata H, Ageyama N, et al (2011) *In vivo* safety and persistence of endoribonuclease gene-transduced CD4^+^ T cells in cynomolgus macaques for HIV-1 gene therapy model. PLoS One 6: e23585. 21858176 10.1371/journal.pone.0023585 PMC3157387

[27] Okamoto M, Chono H, Kawano Y, Saito N, Tsuda H, et al (2013) Sustained inhibition of HIV-1 replication by conditional expression of the *E. coli*-derived endoribonuclease MazF in CD4^+^ T cells. Hum Gene Ther Methods 24: 94–103. 23442049 10.1089/hgtb.2012.131

[28] Lee JT, Yu SS, Han E, Kim S, Kim S (2004) Engineering the splice acceptor for improved gene expression and viral titer in an MLV-based retroviral vector. Gene Ther 11: 94–99. 14681702 10.1038/sj.gt.3302138

[29] Okamoto S, Mineno J, Ikeda H, Fujiwara H, Yasukawa M, et al (2009) Improved expression and reactivity of transduced tumor-specific TCRs in human lymphocytes by specific silencing of endogenous TCR. Cancer Res 69: 9003–9011. 19903853 10.1158/0008-5472.CAN-09-1450

[30] Olah Z, Lehel C, Anderson WB, Eiden MV, Wilson CA (1994) The cellular receptor for gibbon ape leukemia virus is a novel high affinity sodium-dependent phosphate transporter. J Biol Chem 269: 25426–25431. 7929240

[31] Andreadis ST, Brott D, Fuller AO, Palsson BO (1997) Moloney murine leukemia virus-derived retroviral vectors decay intracellularly with a half-life in the range of 5.5 to 7.5 hours. J Virol 71: 7541–7548. 9311834 10.1128/jvi.71.10.7541-7548.1997 PMC192101

[32] Bowerman B, Brown PO, Bishop JM, Varmus HE (1989) A nucleoprotein complex mediates the integration of retroviral DNA. Genes Dev 3: 469–478. 2721960 10.1101/gad.3.4.469

[33] Kildebeck E, Checketts J, Porteus M (2012) Gene therapy for primary immunodeficiencies. Curr Opin Pediatr 24: 731–738. 23073463 10.1097/MOP.0b013e328359e480

[34] Mukherjee S, Thrasher AJ (2013) Gene therapy for PIDs: Progress, pitfalls and prospects. Gene 525: 174–181. 23566838 10.1016/j.gene.2013.03.098 PMC3725417

[35] June CH (2007) Adoptive T cell therapy for cancer in the clinic. J Clin Invest 117: 1466–1476. 17549249 10.1172/JCI32446 PMC1878537

[36] Restifo NP, Dudley ME, Rosenberg SA (2012) Adoptive immunotherapy for cancer: harnessing the T cell response. Nat Rev Immunol 12: 269–281. 22437939 10.1038/nri3191 PMC6292222

[37] June C, Rosenberg SA, Sadelain M, Weber JS (2012) T-cell therapy at the threshold. Nat Biotechnol 30: 611–614. 22781680 10.1038/nbt.2305 PMC6332500

[38] Hoxie JA, June CH (2012) Novel cell and gene therapies for HIV. Cold Spring Harb Perspect Med. doi:pii: a007179. 10.1101/cshperspect.a007179 10.1101/cshperspect.a007179 PMC3475401 23028130

[39] Ciceri F, Bonini C, Stanghellini MT, Bondanza A, Traversari C, et al (2009) Infusion of suicide-gene-engineered donor lymphocytes after family haploidentical haemopoietic stem-cell transplantation for leukaemia (the TK007 trial): a non-randomised phase I-II study. Lancet Oncol 10: 489–500. 19345145 10.1016/S1470-2045(09)70074-9

[40] Porter DL, Levine BL, Kalos M, Bagg A, June CH (2011) Chimeric antigen receptor-modified T cells in chronic lymphoid leukemia. N Engl J Med 365: 725–733. 21830940 10.1056/NEJMoa1103849 PMC3387277

[41] Brentjens RJ, Curran KJ (2012) Novel cellular therapies for leukemia: CAR-modified T cells targeted to the CD19 antigen. Hematology Am Soc Hematol Educ Program 2012: 143–151. 23233573 10.1182/asheducation-2012.1.143 PMC5536093

[42] Restifo NP, Dudley ME, Rosenberg SA (2012) Adoptive immunotherapy for cancer: harnessing the T cell response. Nat Rev Immunol 12: 269–281. 22437939 10.1038/nri3191 PMC6292222

[43] Levine BL, Humeau LM, Boyer J, MacGregor RR, Rebello T, et al (2006) Gene transfer in humans using a conditionally replicating lentiviral vector. Proc Natl Acad Sci U S A 103: 17372–17377. 17090675 10.1073/pnas.0608138103 PMC1635018

[44] Tebas P, Stein D, Binder-Scholl G, Mukherjee R, Brady T, et al (2013) Antiviral effects of autologous CD4 T cells genetically modified with a conditionally replicating lentiviral vector expressing long antisense to HIV. Blood 121: 1524–1533. 23264589 10.1182/blood-2012-07-447250 PMC3587318

[45] Wilen CB, Wang J, Tilton JC, Miller JC, Kim KA, et al (2011) Engineering HIV-resistant human CD4+ T cells with CXCR4-specific zinc-finger nucleases. PLoS Pathog 7: e1002020. 21533216 10.1371/journal.ppat.1002020 PMC3077364

[46] Maier DA, Brennan AL, Jiang S, Binder-Scholl GK, Lee G, et al (2013) Efficient clinical scale gene modification via zinc finger nuclease-targeted disruption of the HIV co-receptor CCR5. Hum Gene Ther 24: 245–258. 23360514 10.1089/hum.2012.172 PMC3609630

[47] Yu SS, Nukaya I, Enoki T, Chatani E, Kato A, et al (2008) *In vivo* persistence of genetically modified T cells generated *ex vivo* using the fibronectin CH296 stimulation method. Cancer Gene Ther 15: 508–516. 18464805 10.1038/cgt.2008.21

[48] Chono H, Goto Y, Yamakawa S, Tanaka S, Tosaka Y, et al (2011) Optimization of lentiviral vector transduction into peripheral blood mononuclear cells in combination with the fibronectin fragment CH-296 stimulation. J Biochem 149: 285–292. 21106541 10.1093/jb/mvq135

[49] Lamers CH, van Elzakker P, van Steenbergen SC, Sleijfer S, Debets R, et al (2008) Retronectin-assisted retroviral transduction of primary human T lymphocytes under good manufacturing practice conditions: tissue culture bag critically determines cell yield. Cytotherapy 10: 406–416. 18574773 10.1080/14653240801982961

